# A new model for the diagnostic assessment services trajectory for neurodevelopmental conditions

**DOI:** 10.3389/fresc.2024.1426966

**Published:** 2024-11-25

**Authors:** Claudine Jacques, Mélina Rivard, Catherine Mello, Nadia Abouzeid, Élodie Hérault, Geneviève Saulnier

**Affiliations:** ^1^ Department of Psychoeducation and Psychology, Université du Québec en Outaouais, Gatineau, QC, Canada; ^2^ Department of Psychology, Université du Québec à Montréal, Montréal, QC, Canada; ^3^ Department of Psychology, Penn State University—Berks, Reading, PA, United States; ^4^ Centre Intégré de Santé et de Services Sociaux de l'Outaouais, Gatineau, QC, Canada

**Keywords:** neurodevelopmental conditions, children aged 0–7, diagnostic services, services trajectory, community-based participatory research, implementation science

## Abstract

**Purpose:**

The Canadian province of Québec faces several issues regarding the accessibility and quality of diagnostic assessment and the efficiency and continuity of evaluation, support, and intervention services for children with neurodevelopmental conditions (NDCs). To address these issues, the Ministry of Health and Social Services mandated a research team to initiate the development of a reference trajectory, i.e., a proposed model pathway based on national and international best practices and research, for the diagnostic assessment of NDCs in children aged 0–7 years.

**Methods:**

The present study focused on the development of a logic model to operationalize the diagnostic services trajectory using a community-based participatory research approach and informed by implementation science. This involved representatives from multiple stakeholder groups (e.g., parents, professionals, physicians, administrators, researchers). Project steps included an analysis of best practices from a literature review on diagnostic trajectories, focus groups and interviews with stakeholders, and a validation process to ensure the appropriateness of the final model.

**Results:**

The integration of existing research and stakeholder input resulted in a logic model for a new diagnostic services trajectory for children aged 0–7 years suspected of NDCs and identified key ingredients that should be present in its future implementation.

**Conclusion:**

The proposed model for a diagnostic services trajectory is expected to address several systemic issues identified previously. Its implementation will need to be evaluated to ensure its sustained focus on the needs of families and its ability to promote their quality of life, well-being, and involvement.

## 1 Introduction

In many countries, evidence of neurodevelopmental conditions (NDCs) is identified too late or goes undetected (1, 2). When a developmental delay is suspected, waiting lists for assessment further compound the problems posed by a lack of timely detection. As a result, appropriate support services are not provided promptly, which can negatively alter the developmental trajectory of children (3, 4). For instance, many children will enter school without having received the necessary supports. This can compromise social integration and academic success (5). Yet, there is consensus regarding the importance of early identification and intervention for all children with developmental challenges (6–8).

At a systemic level, families are faced with problems in accessing high-quality of care (5, 7, 9). Families may not be able to follow a complex bureaucratic procedure to access diagnostic assessment. Residents of remote areas must travel extensively to obtain services without the necessary resources to do so. Even where assessment services are readily accessible, long waiting times, discontinuities during transitional periods (e.g., discontinuity between services), and the complexity and lack of coordination of service systems, along with significant staff shortages, negatively affect the quality of families’ services trajectory (6, 7, 10, 11). These challenges are exacerbated by the lack of guidance and support provided to families as they navigate the system and withstand extensive waiting periods (8, 12–16). As a result, families struggle with the impact of lack of formal support, which exacerbates social isolation and experience of distress (6, 12, 13, 17–20).

At the clinical process level, families face evaluation practices that are not always based on evidence or are not applied according to best practices due to a lack of physicians and specialists, which further accentuate disparities in access to quality services in various institutions (1, 2, 7, 21). Furthermore, the heterogeneity of practices across regions adds to territorial inequalities (8).

At an individual level, the heterogeneity and overlap of clinical profiles within the population of young children with developmental delays further complicate NDC screening, evaluation, and diagnosis (5). Children may present, for instance, ambiguous profiles such as developmental delays (i.e., not reaching the developmental milestones expected for their age) or qualitative deviations from the norm without quantitative delays (22, 23). Also, access to diagnostic assessment and early intervention for children aged six and over is problematic. Generally, this age group no longer has access to early diagnostic assessment services in developmental clinics designed for younger children. A diagnosis may be requested when they enroll in school, which represents an additional burden for families and can hinder access to often limited school-based accommodations and support resources (24). The process for the diagnostic assessment of NDCs in older children is less clearly defined, which further limits the possibilities of accessing appropriate evaluation and intervention resources (25). Taken together, these issues affect access to early intervention and services at the school level. The lack of timely access to support often leads to significant dissatisfaction and distress among parents, who feel unprepared to meet their child's specific needs, and can impede the child's developmental progress (2).

A provincial survey on the development of children in Québec (the Canadian province where the present study was conducted) found a high prevalence of developmental delays at school entry: one in three children had delays in at least one developmental domain [see (5)]. Developmental delays in children are considered a major risk factor for learning and behavioral difficulties in school as well as various psychosocial challenges (e.g., in terms of quality of life, mental health, work integration) throughout life (5, 26). According to the survey, however, many children did not receive services before kindergarten and, thus, had no support before the critical transition to school. This situation motivated the province's Ministry of Health and Social Services (MHSS) to implement the Act Early (in French: *Agir tôt*) program to identify indicators of developmental difficulties prior to school that would help accelerate referrals to early intervention or diagnostic assessment when a NDC is suspected (27). However, as shown by Tardif (28), earlier and better screening practices did not necessarily translate into improved access to intervention services. This issue has led Québec researchers, clinical experts, and service providers to initiate a project to co-create a new model for the NDC services trajectory that would take an integrated care approach to screening, assessment, and support. In doing so, they sought to rethink service delivery to address systemic barriers and inequities in access to evaluation and intervention services. This article presents the final logic model for this new provincial assessment and support services trajectory for NDCs, as co-created through this participatory research approach. In doing so, the article outlines the steps and results at each stage of the model's co-development of the model and demonstrates how data were used to support its development.

The present article sought to summarize the results stemming from a three-stage data collection process leading up to the logic model for a new, integrated diagnostic services trajectory for NDCs. The objective of the first stage was to develop a preliminary logic model based on the scientific literature in the field of diagnostic assessment, including data from large research initiatives conducted in two regions of Quebec to assess approaches to diagnostic assessment services aimed at alleviating systemic accessibility barriers. The second stage sought to adapt this model based on the viewpoints and expertise of several stakeholders (i.e., advisory committee members, parents, clinical experts, and researchers). The objective of the third stage was to finalize the logic model by validating these activities with a subgroup of stakeholders through a final research step of data validation and stakeholder feedback.

## 2 Materials and method

The research project took place between May 2021 and July 2022. The study protocol was approved by the research ethics committee of nine healthcare centers: Centre intégré universitaire de santé et de services sociaux de la Mauricie-et-du-Centre-du-Québec (CIUSSS MCQ), CIUSSS du Bas-Saint-Laurent, CIUSSS du Saguenay-Lac-Saint-Jean, CIUSSS de l'Estrie-CHUS, Centre intégré de santé et de services sociaux (CISSSS) de Chaudières-Appalaches, CISSS de la Montérégie-Centre, CISSS des Îles, Centre Hospitalier Universitaire (CHU) de Québec Université Laval et CHU Sainte-Justine and by the Université du Québec à Montréal and Université du Québec en Outaouais.^1^

### 2.1 Three frameworks as anchors to the development of the services trajectory

The research team and stakeholders co-developed the logic model for the new diagnostic services trajectory based on three frameworks: (1) community-based participatory research [CBPR; (29)]; (2) planning stages as defined by Chen (30, 31); (3) pathways as defined by Vanhaecht et al. in relation to the concept of services trajectory (32). According to the latter, a service pathway for a specific population encompasses the following elements: explicit objectives and evidence base; integration of user expectations; communication and coordination mechanisms; sequence of the multidisciplinary care team's activities with patients and their families; documentation, monitoring, and evaluation of gaps and results; and identification of relevant resources for the implementation and operation step [free translation; (33)]. With these three frameworks in mind, we constructed an extraction grid using the concept of logic model as a visual representation to structure and describe the theory of a program, here, a trajectory (30, 34). This grid included the following major components of a logic model as illustrated in Supplementary Appendix A: problem, objectives, target groups and groups responsible, activities, inputs, outputs, outcomes and influencing factors [see (103) for definitions of these components]. This framework guided data collection and analysis throughout the three stages: (1) analysis of best practices and research, (2) focus group and interviews with stakeholders, and (3) data validation and stakeholder feedback of the research project by supporting the triangulation of empirical, professional, and experiential knowledge regarding best practices in diagnostic assessment.

### 2.2 Phases of the research project

#### 2.2.1 Planning phase: the advisory committee

Consistent with CBPR principles, all steps and guiding principles for the research project were co-decided by an advisory committee composed of specialists, physicians, and administrators. The first two authors were invited by a project manager from the MHSS to participate in this committee, which included stakeholders from nine out of the 17 establishments within Québec's public health and social services system and the National Institute for Excellence in Health and Social Services of Québec. The mandate of the working group meetings was to iteratively determine the principles and stages of the research project needed to support the development of the reference trajectory for diagnostic evaluation. Reaching a consensus on the guiding principles and planning the stages of the research required 12 meetings and several e-mail exchanges of meeting notes summarizing the topics addressed and discussions and outlining the responsibilities and tasks of committee members between 2019 and 2021 [see Héreault et al. (104)].

### 2.3 Data collection phase

Data collection took place over three stages: (1) analysis of the finding of a large research initiative conducted in two regions of Quebec to assess innovative diagnostic services trajectory aiming to alleviate systemic barriers to access support for families and scoping review (2) stakeholder consultation, and (3) validation and development of the final model.

#### 2.3.1 Instruments and procedure (phases 1–3)

##### 2.3.1.1 Creating a first model with pilot projects and scoping reviews

The first stage of the study took place from May to November 2021. The research team analyzed four sources of data. The first two sources were central data sources derived from the two previously mentioned large research initiatives conducted to assess innovative diagnostic services in Quebec (here after pilot) [(5, 105, 106, 107, 108–110), see also https://chaireditc.uqam.ca/vcmf/]. Other secondary sources of data were two scoping reviews on factors influencing parental satisfaction with respect to the NDC diagnostic services trajectory (35)^2^ and one on the experience of parents of young children with developmental delays with their child's diagnostic assessment process.^3^

Seven analytical steps were used iteratively to develop the logic model for the NDC diagnostic services trajectory. First, three research team members (CJ, MR, and ÉH) created an extraction grid (including the major components of a logic model). Second, they extracted the data from the two pilot projects to create a model for each project. Third, ÉH consolidated the extractions to produce a document summarizing the information from each project according to the extraction grid. Consensual data were grouped together, while non-consensual data were excluded. Weekly meetings were held between the three members to ensure a shared understanding of the information included in each logic model. Fourth, two research assistants trained in implementation science (CB and MM) reviewed the two models. The aim of this step was to ensure the quality and validity of the information included in the models by involving individuals who were not part of the initial extraction process but were already familiar with the projects studied. All modifications were discussed with the three research team members. Fifth, the models were then combined into a single model by one team member (ÉH). Information was synthesized where necessary, duplicates were removed, and non-consensual data were retained to highlight discrepancies related to contextual specificities. Sixth, ÉH made modifications to the logic model to include information from the scoping reviews. By the end of this stage, all the information gathered from the two pilot projects and the two scoping reviews had been integrated. Finally, the resulting logic model was revised by the three team members to produce a first version of the logic model (including its components and associated themes) for the NDC diagnostic services trajectory. This stage involved compiling information, reformulating statements, and ensuring that the synthesized information was consistent with the research project's mandate and objectives.

##### 2.3.1.2 Creating a second model with stakeholders’ involvement

For the second stage, a video lasting approximately 45 min. introduced stakeholders to the activities identified in the preliminary logic model (see Supplementary Appendix B). An accompanying document that synthesized the activities of the logic model was provided to participants. The stakeholders shared their experiential, professional, and scientific views and knowledge in semi-structured individual interviews and focus groups. Two of the authors conducted these interviews through a videoconferencing platform. Clinical experts and researchers were interviewed individually (approx. 60 min.). Parents and advisory committee members were interviewed in a focus group lasting approximately 90 min.

##### 2.3.1.3 Creating the final model: validation process with the stakeholders in charge of its implementation

For the third and final stage of data collection, the advisory committee participated in a data validation exercise to ensure the accuracy of the final model (36, 37). Committee members consulted a document summarizing the two previous phases and explaining the third phase and another document that described the activities of the final model (see Supplementary Appendix C). They were asked to indicate whether they agreed or disagreed with each activity within the final model and to propose adjustments in a feedback form.

#### 2.3.2 Participants (stage 1)

The first two authors participated in this stage, along with the research coordinator (ÉH) and two research assistants who were familiar with each of the projects selected by the MHSS (CB and MM).

#### 2.3.3 Participants (stages 2 and 3)

Three groups participated in the second stage of the study: (1) the advisory committee, which included professionals, physicians, and administrators working in the field of diagnostic assessment (*n* = 9), (2) parents whose child was diagnosed with a NDC before the age of 7 (*n* = 9) and (3) clinical experts and researchers (*n* = 11) in the field of NDC assessment. Inclusion criteria for this stage were (1) living in Quebec and (2) speaking French and, for parents specifically, (3) having a child with an NDC diagnosis made after April 2017 (i.e., denoting a recent experience with assessment services). Parents and clinical experts and researchers were selected through a purposive sampling method to achieve a diversity of participant profiles. Members of the advisory committee were identified by the MHSS. Table 1 summarizes the clinical and sociodemographic characteristics of parents. Table 2 outlines the areas of expertise and professional or physician designations of the two other groups. The members of the advisory committee were involved in the third stage of the study.

**Table 1:** Clinical and Sociodemographic Characteristics of Parents.

Characteristics	*n*
Relationship to the child	
Father	1
Mother	8
Age	
18–35 years	1
36–45 years	4
46–55 years	4
Country of birth	
Canada	8
Other	1
Language spoken at home (with the child)	
French	9
English	5
Other	0
Number of people in the household (including the parent)	
3 persons	4
4 persons	2
5 persons	2
6 persons	1
Net annual household income	
Under CAN$19,999	1
CAN$20,000–39,999	0
CAN$40,000–59,999	3
CAN$60,000–79,999	1
CAN$80,000–99,999	2
Over CAN$100,000	2
Child's gender	
Male	7
Female	2
Child's age at diagnosis	
3 years	2
4 years	3
5 years	4
Child's diagnosis(es)	
Verbal dyspraxia	1
Visual-spatial dyspraxia + CDD + ADHD	1
GDD	1
GDD + Language Disorder	1
Tourette's syndrome + ADHD + ASD	1
Language disorder + ADHD	2
ASD	2
Age of the child at the time of the study	
6 years	1
7 years	1
8 years	4
9 years	2
10 years	1

CDD, childhood disintegrative disorder; ADHD, attention deficit; GDD,  global developmental delay; ASD, autism spectrum disorder (APA, 2013).

**Table 2:** Areas of Expertise and Professional Designations of the Advisory Committee.

Expertise and professional designations	*n*
Advisory committee members and clinical experts	
Speech therapist	3
Pediatrician	2
Developmental pediatrician	2
Child psychiatrist	1
Psychiatrist	1
Psychoeducator	3
Psychologist	1
Psychologist (private services)	1
School psychologist	1
Researchers	
Neuropsychology	1
Speech therapy	1
Psychology	1
Nursing	1
Social work	1

#### 2.3.4 Data analysis (stages 2 and 3)

Interviews were transcribed by a research assistant and subjected to a mixed deductive and inductive content analysis (38, 39). First, *directed content analysis* was used to analyze data through the application of deductive categories, which is useful for validating a theoretical framework (40). Second, *summative content analysis* enabled the identification, quantification, and interpretation (41, 42) of specific data to analyze comments from the stakeholders about the completeness, accuracy, and quality of the logic model. Two research assistants used NVivo 12 (43) to extract these comments (as “comment units”) from data obtained on the model. Then, three research assistants analyzed the comment units according to a coding grid (created and reviewed by the research team) that included: (1) activities “needed clarification” that needed additional details or rephrasing to be fully understood, (2) “missing” activities that were absent from the presented model and that stakeholders felt were necessary, and (3) activities that “do not meet needs.” The comment units were then grouped and categorized to identify what should be clarified, added, or removed from the description of activities in the model. Finally, in a reflective work session, the five authors discussed how to incorporate these categories into the model's activities. These were considered for inclusion based on three criteria: (1) triangulation of the theme across participant groups, (2) consistency with the research project's mandate and objectives, and (3) consensus within the research team regarding relevance to the concept of “activities” in a logic model (See Supplementary Appendix D for a representation of the iterative, reflective work process).

In the third stage, validation, participants’ agreement on the activities in the final model was computed. Qualitative comments in the feedback form were reviewed by the research team and facilitated the revision of activities that required further clarification. Two research assistants and two authors analyzed the advisory committee's comments to identify their overall perspectives on, and their perception of facilitators and barriers within, the trajectory proposed in the final iteration of the model.

## 3 Results

### 3.1 Planning phase

The advisory committee appointed the first two authors to develop and lead a research project focused on the identification of evidence-based practices described in the scientific literature and evaluated in the Quebec context. These practices were intended for implementation in a diagnostic evaluation trajectory for children suspected of having a NDC, were to be drawn from the two pilot projects and the scoping reviews selected by the advisory committee.

The advisory committee reached a consensus on nine principles to guide the development of the research project. Specifically, they determined that it should (1) be based on best practices (research) that frame the assessment of young children; (2) include an assessment of the child's and family's service needs and not only diagnostic assessment; (3) include family support services outside of diagnostic assessment and be part of an evaluative trajectory perspective that includes screening, family support, and response to intervention; (4) be built on a collaborative approach that recognizes and integrates the knowledge of various stakeholders; (5) address documented issues related to timely access to care and services and to the continuity and quality of such care and services; (6) include the assessment of all children with developmental delays and atypicalities (i.e., not limited to intellectual disability and autism); (7) meet the needs of children aged 0–7 years 11 months (i.e., not 0–5 exclusively) to address issues of access to diagnostic services surrounding the transition to school; (8) aim to improve satisfaction and the quality of the experience for stakeholders, especially families; (9) allow sufficient flexibility for organizational trajectories to adapt to the regional particularities of the various centers in Quebec [see also (103) for the detailed collaborative process which led to the development of the research protocol].

### 3.2 First stage

Eight components of the logic model were identified through the analysis of the pilots and review sources. This yielded a preliminary model for a diagnostic services trajectory that included these components and their respective themes (see Supplementary Appendix E). The Problem component, i.e., the context explaining the need to develop a reference trajectory for the organization of care and services, included four issues. The Objectives component, which aims to respond to the issues raised in the Problem, was summarized into four main goals. Two groups emerged in the Target Groups component and two themes in the Groups responsible component. Four themes were identified in the Inputs component, which refers to the resources required to carry out activities. The Activities component describes the practices to be implemented to achieve the objectives and results within the framework of a services trajectory (refer again to Supplementary Appendix C, which lists the first iteration of the Activities component of the logic model that was presented to participants in the second stage). Six subcomponents were used to categorize Activities (see Supplementary Appendix F). These distinguished between activities occurring before, during, and after the diagnostic assessment: three chronological subcomponents (pre-assessment, diagnostic assessment, and post-assessment) and three transversal (non-chronological) subcomponents that connected the chronological subcomponents.

Nine themes emerged for the Outputs component, which can demonstrate whether activities were carried out as planned. Ten themes emerged from the analysis of statements related to the Results component. Finally, 87 barriers to, and 73 facilitators of, the completion of logic model activities as prescribed were identified as part of the Influencing factors component. Supplementary Appendix G details the barriers and Supplementary Appendix H the facilitators. These factors can have either negative or positive impacts on the quality of implementation and the achievement of expected results for each component of the logic model. In transversal component, several barriers or facilitators have an impact various elements grouped under nine themes: access to services, physicians and specialists’ expertise development, interprofessional collaboration, availability, stability, and distribution of resources, continuity of services provided throughout the trajectory, parents’ collaboration with physician and specialists, information-sharing between referrers, assessment services, and intervention services, evaluation of the implementation and effects of the trajectory, optimal implementation of a reference trajectory on a provincial scale. In pre-assessment component, one barrier or one facilitator have impact various elements grouped under one theme: quality of screening. In the diagnostic assessment, several barriers have impact various elements grouped under two themes: quality of the assessment process, access to assessment services. Similarly, facilitators have impact elements related to quality of the assessment process. In post-assessment component, several barriers or facilitators have impact various elements grouped under two themes: quality of post-assessment interventions, efficiency of post-assessment services.

### 3.3 Second stage

The stakeholder consultation stage primarily focused on the Activities component of the logic model and prompted modifications to the description of activities included in the preliminary model. Stakeholders also expressed opinions on other components. This feedback was also used to improve the model.

Based on the three criteria for inclusion in the logic model mentioned previously (i.e., triangulation, consistency, and consensus), some stakeholder comments were retained and led to revisions of pre-assessment (*n* = 15), diagnostic assessment (*n* = 25), post-assessment (*n* = 6), and transversal (*n* = 18) activities. Among the pre-assessment activities that needed clarification (*n* = 5), stakeholders stressed the importance of correctly characterizing the population targeted by the trajectory. A member of the advisory committee said: “*Write 0–7 years, instead of early childhood, as [early childhood] refers to 0–5 years.”* Under missing activities (*n* = 7), they emphasized the importance of determining the level of support. A parent shared, “*Moreover, before the “one-stop shop”, there perhaps needs to be a form of triage, because there are people for whom going directly to a psychologist can be a good thing, while others should rather go to a multidisciplinary center.”* Under activities that did not meet needs (*n* = 3), they identified the need for a key player present throughout the trajectory, as pointed out by a member of the advisory committee: “*the absence of a key player at the time of screening.”*

Among the diagnostic assessment activities that needed clarification (*n* = 10), stakeholders asked to define the referral process for complex cases. An expert said: “*[…] referring children with more complex profiles. But more complex than what, you know? It depends on who you talk to.”* In relation to missing activities (*n* = 8), they pointed out the importance of having a centralized information system. An expert suggested: “*when we talk about receiving, reading, and making an initial analysis of the case file, that’s fine, and then acknowledging receipt to the referent environment, well, why not have a dashboard that would also enable parents to follow the evolution of the situation?”* Under activities that did not meet needs (*n* = 7), they mentioned the fact that the services are not tailored to needs. A member of the advisor committee stated: “*give the diagnostic assessment according to need, and that speaks to me a lot, because what we see isn’t necessarily based on need, it's based on available resources. So, sometimes, we’ll say “okay, there's a real need for speech therapy, but there's a six- or seven-month wait, so okay, we’ll offer such and such a service instead’ when that's not what needs to be done, it's not the trajectory, it's not the child's needs, so to go according to needs, that speaks to me, you know, it's something important.”*

In the post-assessment phase of the trajectory, under the activities that needed clarification (*n* = 1), a stakeholder indicated the importance of identifying the appropriate program according to the child's needs. The expert posed the following question: “*in complex situations where the service to be rendered is not evident, and where, for certain elements of the diagnostic assessment, the question also arises of what to prioritize.”* Under missing activities (*n* = 5), stakeholders stressed the need for post diagnostic support measures while waiting for specialized intervention services. A member of the advisory committee who specialized in autism suggested: “*We could have different forms of support from the assessment team, depending on the child's needs, while waiting for services to be put in place.”* There were no comments about post-assessment activities that did not meet needs.

With respect to transversal subcomponents, under activities that needed clarification (*n* = 8), stakeholders emphasized the need for continued support and training. A parent said: “*From that point of view too, I think training would be important, you know? To be able to help providers disclose it? To talk with the parent.”* In terms of missing activities (*n* = 9), stakeholders proposed the addition of a linguistic and cultural interpreter. An expert stated: “*There's a translation, a cultural accompaniment. The professional must learn what they mean when they use a particular word, and what it means to the parent. Conversely, the parent needs to understand what the clinician is saying. We need this kind of linguistic interpreter, and culture needs to be more present than we’ve seen before.”* Under activities that did not meet needs (*n* = 1), a stakeholder pointed out the necessity to consider the clinical profile category. The expert expressed: “*How can we do that, you know, have trajectories that consider all possible scenarios? We can’t. So you have to go by cluster, you have to go, it seems to me, by large portraits. Well, at the same time, that's the way it is now, in the sense that everything is too specialized. I don't think that's the way to go.”*

Finally, stakeholders commented on the overall value of logic model. The research team identified six key characteristics of the proposed reference trajectory: it is comprehensive, consistent with the ministerial Act Early program, solution-oriented, it integrates multiple expertise and viewpoints (scientific, professional, experiential, parental), it is child-centered within an ecosystemic perspective, and supports parents and promotes their involvement (see Supplementary Appendix I).

### 3.4 Third stage

#### 3.4.1 Advisory committee assessment and activities of the final logic model

The validation process deployed in the third and final stage enabled the advisory committee to identify potential facilitators and barriers to the implementation of the trajectory. These factors were grouped into eight categories that would need to be considered in the implementation of the new trajectory: administrative support; presence and role of key players; availability of human and physical resources (in particular, digital technology); assessment and intervention approach based on needs rather than diagnosis; coordination of public and private services; promoting functioning and providing information on the diagnostic trajectory (particularly among parents, but also health, social service, and education professionals); promoting reserved acts, i.e., acts for which practice is regulated by a professional order or college; and continuing education and development of professional expertise; (see Supplementary Appendix J).

The feedback shared by the advisory committee confirmed the validity of the proposed activities: agreement on the statements relating to the subcomponents of activities (44) averaged 96.6%. Among the eight participants for this phase, agreement exceeded 95%: three members agreed with all statements (100.0%), four members agreed with 66 statements (98.5%) and one member agreed with 64 statements (95.5%). The other two members also presented high (above 85%) levels of agreement in relation to the activities presented. Supplementary Appendix K presents the data from the validation phase for each activity.

#### 3.4.2 Final logic model and agreement

In the transversal activities subcomponent, six elements were identified as needing to be implemented on an ongoing basis throughout the trajectory: (1) the involvement of parents as partners in the services trajectory (95.0%); (2) the accompaniment of the child and their family, from the beginning, by a key player who ensures that they are directed to the individualized resources they need (90.0%); (3) accessibility for all children and families living in Quebec (100.0%); (4) adaptation to the evolving needs of children and their families (100.0%); (5) support for stakeholders to carry out coordinated and concerted actions with children and their families (98.8%); and (6) the evaluation of the quality and effects of the implementation of the trajectory across the various institutions (100.0%).

Pre-assessment activities included activities organized chronologically according to three themes addressed: (1) monitoring (100.0%); (2) referral for screening or diagnostic assessment (96.7%); and (3) screening, in case of a referral (95.0%).

Two activities were transversal to the pre-assessment and diagnostic assessment subcomponents (i.e., these can be performed at any time, as needed, during these two stages). The two themes were (1) accessing support and services before a formal diagnosis is made (100.0%) and (2) listening to parents and their concerns (100.0%).

Activities in the diagnostic assessment subcomponent were grouped under four themes: (1) reception and analysis of the case file (94.0%); (2) functioning of the interprofessional team (98.0%); (3) expertise and continuing education of physicians and specialists (100.0%); and (4) diagnostic findings (93.3%).

The transversal activities common to the diagnostic assessment and post-assessment subcomponents were grouped under a single activity: linking diagnoses and services (93.3%).

Activities within the post-assessment subcomponent, which related to support and services following a diagnosis, were organized chronologically into three themes: (1) monitoring of providers who provide intervention services to the child (100.0%); (2) monitoring of providers who work with the child (100.0%); and (3) monitoring and support for the child, parents, and family (100.0%). Refer again to Supplementary Appendix C which presents the final version of the resulting logic model and Supplementary Appendix L presents a visual representation of the activities of the logic model.

## 4 Discussion

Using a transformative and collaborative research method, we proposed an innovative way to address long-standing and complex accessibility issues in diagnostic assessment and early intervention for children with developmental delays or atypicalities (45, 46). In collaboration with a diverse group of stakeholders (parents, professionals, physicians, administrators, clinical experts, and researchers), we developed a logic model for a new diagnostic services trajectory for children aged 0–7 years suspected of having an NDC.

We used an adaptation of Chen's framework (30) to build the logic model for a new services trajectory in three stages. The first stage organized and synthesized empirical data on best practices in diagnostic assessment for this population. The second stage integrated stakeholders' perspectives on the preliminary logic model derived from the initial literature review. Their feedback indicated that they viewed the model as appropriate and comprehensive, but also included suggestions that led to an improved version of the model. The final phase involved a validation exercise with the initial advisory committee to review the Activities component of the logic model. This phase explored the validity, relevance, and feasibility of the activities to be included in the proposed trajectory (47). This step enabled the advisory committee to influence the choices made by the MHSS through a series of contextualized recommendations. This multi-stage process supported iterative cycles of stakeholder participation throughout the project while respecting their time and expertise (48, 49).

In addition to developing a comprehensive model for a new assessment and support services trajectory for children aged 0–7 years suspected of having a NDC, the stakeholders identified key ingredients (50) that should be present in its implementation. They specifically emphasized the importance of a key player, continuity in the services trajectory, transversal activities, a family orientation, training for specialists and physicians, and ongoing evaluation.

### 4.1 The presence of a key player involved with families for the continuity of services

Stakeholders firmly believed that the presence of a key player (navigator) is essential to guarantee the quality of the services trajectory and enhancing support for families. They highlighted that the presence of this key player from the beginning of the trajectory and the continuity of their involvement with the family were important elements. This perspective is supported by the scientific literature (19, 51) and ministerial guidelines (52, 53). While this concept has been implemented in health care services, this is not the case for the trajectory for children with suspected NDCs. Presently, there are no key players within NDC assessment teams to whose role is to support families. Issues related to role definition but also to resources and training (54–56) could influence the ability of organizations to deploy this core activity of the logic model. Consequently, to ensure that this practice is effectively implemented, it would be important to better define the role of key player and the ways in which it could be actualized according to evidence-based practices for this specific population (57, 58). Stakeholders identified essential elements that must be associated with the function of the key player that are in line with the literature, e.g., relational closeness, a support and wellness orientation (59).

### 4.2 An integrated diagnostic assessment services trajectory

A second key ingredient repeatedly raised by stakeholders throughout their participation concerned the importance of considering the various services upstream and downstream of the assessment process. According to them, these different phases, and all the activities they imply, are inseparable. Therefore, they must be carried out in continuity and integrated into a single process. Improving children's and their families' experience will require the development of organizational trajectories for the various service segments that articulate with each other. This relates to the importance of the coordination, accessibility, continuity, and flexibility of services (60–65). Stakeholders stressed the importance of designing a trajectory for children suspected of having a NDC that integrates monitoring and screening, post-diagnostic services, and interdisciplinary services. This approach would enable early access to intervention and ensure the continuity of interventions right up the child's transition to school, thus promoting equal opportunities as recommended by the World Health Organization (WHO) and the United Nations Children's Fund (UNICEF) and supported by numerous guidelines (5, 44, 66–68). Diagnosis should not be the ultimate goal of the trajectory. It should be viewed as a tool to better understand the characteristics, challenges, strengths, and needs of the child and their family (69–72).

### 4.3 Family-centered services trajectory

Stakeholders emphasized the importance of including cross-functional activities to meet families' needs from the beginning of the trajectory and on a continuing basis. These family-oriented activities can take various forms, such as support for navigating service systems, information on diagnoses and services, training on practices to adopt at home, and support related to psychological health and family adjustment (7, 19, 73). The importance of ongoing support for children and families throughout the trajectory is a major element of our research. Families have considerable needs from the moment they suspect delay or atypical development in their child (74–76).

The parents who participated in the co-creation of the logic model comprehensively explained what the presence of this type of support could facilitate and the economic, social, and emotional consequences of its absence. This is one example of their significant contribution to the present research project and demonstrates that a trajectory cannot be designed, implemented, and evaluated without the active involvement and ongoing collaboration of parents [for the importance of partnering with families see (77–79)]. The two focus groups we held with parents reflect our research team's emphasis on listening to and considering their perspective. Their testimonials brought to light facilitators and barriers experienced in contact with services along with concrete measures that could facilitate the fluidity of services and better meet the needs of their children and families. Moreover, the involvement of parents in the present study led to a new research project to develop a web portal to provide information and support to families. This portal is also being co-developed, according to participatory methods, with parents to ensure it meets the needs they identified (80, 81).

### 4.4 Training and support for teams involved in the trajectory

During the validation stage, members of the advisory committee underscored their desire to receive more training to acquire in-depth knowledge of atypical as well as typical development and of empirically-based evaluation methods. They also wished for more training to develop the clinical skills needed to conduct successful family encounters (e.g., relational closeness), to recognize appropriate ways to communicate diagnostic findings, or to promote family adjustment during difficult times. Implementing an integrated services trajectory for children suspected of having a NDC based on best practice requires qualified teams with a high level of knowledge (82–85). To achieve this, all service providers must have access to ongoing training. Training and team support were two key elements raised by stakeholders and supported by evidence (77, 86–88). These would not only improve the quality of children's and families' experience, but also increase feelings of competence and job satisfaction, and reduce distress and burnout among providers (89). The integration of continuing education opportunities is essential to adapt to the needs of stakeholders and to new knowledge and evidence-based practices emerging from scientific literature. Our work reflects the need for institutions to put in place various forms of training, supervision, and support as they seek to improve the quality of services provided by those conducting assessments and interventions.

### 4.5 Importance of ongoing evaluation

One last key ingredient stemming from stakeholder feedback was a continuous process of evaluating and improving the reference trajectory in various organizational contexts. The continuous evaluation of practices, including innovations or organizational changes, is prescribed by professional corporations (87, 90–92). The improvement of organizational trajectories (see Redacted for blind review) will also have to be dynamic in the sense that the relationships between the various components will have to be considered by organizations (e.g., the inputs that have an impact on the compliant performance of activities) (47, 93–95). The deployment stages of organizational trajectories must consider change management, particularly regarding implementation and the adjustments required (96–98). Finally, the process of developing and implementing the reference trajectory and organizational trajectories should be iterative (86, 99) and supported by program evaluation initiatives. Program evaluation should include diverse stakeholders in the reflections and decisions surrounding the research process and in data collection (91, 100–102).

### 4.6 Limitations of the present study

Significant delays in terms of ethical approvals, which had to be obtained from eight institutional review boards, had an impact on the implementation of the research project. Consequently, although the information gathered from the three groups proved to be very rich, we lacked the time to adopt multiple data collection methods to suit the different respondent profiles, which is an important element in collaborative research (47). To gain a deeper understanding, it would have been desirable to, e.g., vary participation modalities and levels by conducting individual interviews or focused discussions with each stakeholder group with more flexible scheduling. In addition, although it was justified to conduct the validation exercise with the advisory committee given its involvement from the outset of the project, it would have been interesting to deploy a similar feedback phase with each stakeholder group. This would serve to validate the advisory committee's appraisal, but also to enrich and contextualize the model with input from parents and other experts. Finally, lack of time also limited our ability to reach out to all professions and specialties involved in diagnostic assessment, therefore, e.g., occupational therapists were not included in our sample.

## 5 Conclusion

This study supported the development of a new reference trajectory for diagnostic assessment services for children suspected of having NDCs. Importantly, it also proposed a method for modeling services trajectory that combines rigorous research and the participation of people concerned by these services. It thus advanced our knowledge on the best ways to support families during this crucial moment. We believe that systems of care can only be improved to become more responsive of the needs families when parents, physicians, specialists, administrators, and scholars work together. Although the process we used was intended to guarantee the validity of the outcome, only a comprehensive evaluation of the implementation of the reference trajectory can confirm its fidelity and sustainability.

## Data Availability

The original contributions presented in the study are included in the article/Supplementary Material, further inquiries can be directed to the corresponding author.

## Footnotes

^1^The CISSS and CIUSSS provide services to the population of Quebec's 16 territories. They are points of reference where people can turn for help with health and psychosocial problems. The population can receive appropriate services there, or be referred to another resource forming part of the territorial service network. Other establishments in the health and social services network (4 university hospital centers and 3 university institutes) offer specialized and superspecialized services beyond the boundaries of the region to which they belong, including the CHU-Ste-Justine.

^2^Pérez CH, Rivard M. *Évaluation et satisfaction des parents à l'égard de la trajectoire de services diagnostiques de jeunes enfants ayant un trouble neurodéveloppemental* (unpublished bachelor's thesis). Université du Québec à Montreal, Montreal (2021).

^3^Lacelle C, Dykstra-Légaré T, Saulnier G, Jacques C. The experience of parents of young children with developmental delays in the process of diagnostic assessment and intervention: A scoping review. (in preparation)
