# Supplementary material for: A new model for the diagnostic assessment services trajectory for neurodevelopmental conditions
Source: Front Rehabil Sci. 2024 Nov 25;5:1426966. doi: 10.3389/fresc.2024.1426966 (PMC11625808; doi:10.3389/fresc.2024.1426966)
Supplement: Supplementary file 1 [file Datasheet1.pdf]

Appendix A

Main Components of a Logic Model

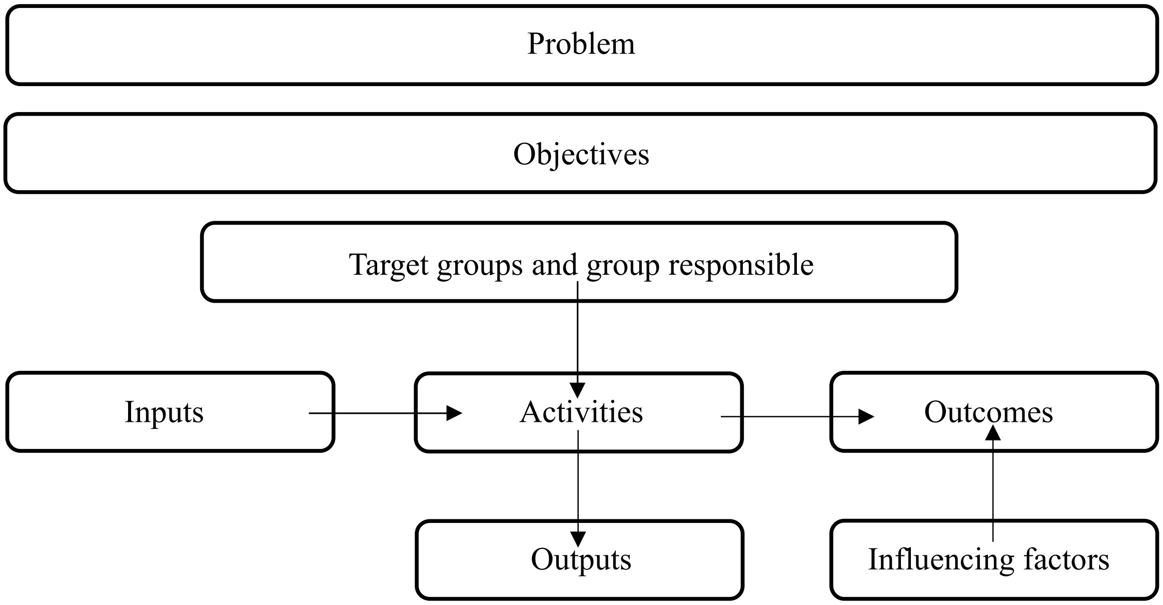

Appendix B

First Iteration of the Activities Component of the Logic Model

Activities Prior to Diagnostic Assessment (Pre-Assessment)
Monitoring
1. Set up a universal monitoring system that 1) reaches all families; 2) involves all early childhood partner organizations; 3) empowers individuals involved in early childhood intervention to identify signs of delays or developmental atypicalities; and 4) empowers individuals involved in early childhood intervention to orient refer to screening/or diagnostic assessment services.
2. Convey information to individuals involved in early childhood intervention about 1) identifying children who show signs of developmental delays or atypicalities; 2) best practices to disclose concerns to parents; 3) mechanisms to request a screening referral; and 4) mechanisms to keep parents informed of the current and future process.
Referral for Screening
3. Implement a referral system that enables early childhood partners to refer for screening or diagnostic assessment services at any time.
4. Implement a single access point for screening that contributes to a better fluidity in the processing of requests.
5. Ensure that early childhood partners identify their criteria for referral to screening or diagnostic assessment services.
Screening
6. Implement a screening system for children at risk of developmental problems for whom concerns have been raised by a parent or caregiver. The aim of this system is to identify children's needs so that they can be directed rapidly to appropriate services and a more in-depth diagnostic assessment.
7. Develop a mechanism to communicate screening results and future steps to families.

Diagnostic Assessment Activities
Case Intake and Analysis to Establish the Assessment Trajectory
8. Identify eligibility criteria for diagnostic assessment services, considering the fact that clinical profiles vary among children and may evolve during early childhood.
9. Receive, read, and make an initial analysis of the case file, then acknowledge receipt to the referring environment.
10. Integrate previously collected information.

Diagnostic Assessment Activities *(continued)*
Case Intake and Analysis to Establish the Assessment Trajectory *(continued)*
11. Provide parents with information and questionnaires to complete.
12. Conduct a telephone interview to confirm the reason for referral and the information on file (especially in questionnaires).
13. Establish the diagnostic services trajectory at an interprofessional team meeting according to different pre-established trajectory models to reduce waiting times for families.
14. Refer children with more complex clinical profiles to specialized services, especially when multiple diagnoses, genetic syndromes, and neurological or physical health problems are suspected.
15. Plan the essential and optional stages of the diagnostic assessment according to the needs of the child and their family.
16. Involve the required specialists in the diagnostic assessment, according to the proposed plan.
17. Ensure that the diagnostic assessment is based on the clinical judgment of skilled specialists to analyze and triangulate various measures and sources of information.
18. Base diagnostic assessment on 1) standardized, norm-referenced and non-standardized measures; and 2) multiple data sources documenting the child’s functioning in their various living contexts (e.g., home, daycare, school) and according to various respondents (e.g., parents, educators, teachers).
19. Make a global assessment of all spheres of the child's development.
20. Consider 1) family situation; 2) situations in the child's other living environments; 3) the child's antecedents (i.e., developmental history); and 4) the current clinical picture of the child.
Functioning of the Interprofessional Team
21. Define and distinguish the mandates of the specialists involved in assessment, taking into account professional corporations, to promote understanding and respect of their mandates and responsibilities.
22. Structure a form of interprofessional collaboration enabling members of specialist groups to take collective action towards a common goal, through a process of communication, reflection, decision-making, intervention, and learning.

Diagnostic Assessment Activities *(continued)*
Functioning of the Interprofessional Team *(continued)*
23. Organize regular meetings between specialists to discuss clinical impressions and draw collective diagnostic findings.
24. Involve all specialists whose activities can contribute to diagnostic assessment.
25. Adapt the level of interprofessional collaboration to the complexity of the situation.
Diagnostic Findings
26. Consult parents before the assessment meeting to determine the support they will need when the diagnosis is disclosed.
27. Include in the assessment meeting: 1) disclosure of the diagnosis or clinical impressions; 2) presentation of the results of information gathering; and 3) presentation of recommendations and information based on the needs of the child, their family, and their living environments.
28. Ensure that the assessment meeting is conducted by specialists already involved in the assessment who apply good practices to develop and consolidate a close relationship with parents.
29. Include in the report: 1) assessments conducted by all specialists and 2) recommendations based on the needs of the child, their family, and their living environments.
Post-Diagnostic Support and Services (Post-Assessment)
Monitoring of Organizations Offering Specialized Services to Children
30. Establish communication mechanisms between diagnostic assessment and post-diagnostic intervention settings to determine which services are best suited to the child's specific needs.
31. Refer to appropriate services or intervention programs, according to the needs of the child and their family identified during diagnostic assessment and according to best clinical practices.
Monitoring of Environments Attended by the Child
32. Follow up with the child's caregivers to inform them of the child's characteristics and provide them with intervention strategies.
Monitoring and Support for Parents
33. Offer, following the assessment meeting, post-diagnostic follow-up meetings with the parents or child, depending on the nature of their needs (e.g., suitable frequency and duration, individual or group, direct or indirect).

Post-Diagnostic Support and Services (Post-Assessment) *(continued)*

Monitoring and Support for Parents *(continued)*

34. Ensure that post-diagnosis follow-up meetings 1) enable parents to ask questions; 2) provide information adapted to the needs of children and parents (e.g., on resources available in the community, on the child's diagnosis and characteristics, on intervention strategies that the child's parents and environments can apply, on specialized programs and services); 3) support parents in filling out financial and material assistance forms (e.g., government allowances, grants from non-profit organizations or charities); and 4) support or refer parents to resources to ensure their psychological well-being.

Listening to Parents

35. Take into account the nature of the needs and the context of the establishments (e.g., geographic, health, human resources) in the choice of modality (virtual or in-person).

36. Take into account parents' priorities regarding their child's development.

37. Map offered services.

38. Identify and advertize post-diagnostic services and support available in the area and their eligibility criteria.

Activities Transversal to Pre-Assessment and Diagnostic Assessment

Pre-Diagnostic Support and Services

39. Implement support and services that meet children's and their families' needs and are accessible at all times, e.g., transdiagnostic support, parent coaching programs, psychological health support, information sharing (on financial support, community services, etc.).

Listening to Parents

40. Consider parents' concerns about their child's development.

Activities Transversal to Diagnostic Assessment and Post-Assessment

Linking Diagnoses and Services

41. Ensure that there is a link between the diagnosis made during assessment and the services offered by the facility so that the diagnosis leads to services.

42. Consider the child's response to intervention as a dynamic process, from the moment of assessment and throughout the developmental trajectory.

43. Ensure that the choice of individualized services is based on assessment results.

Activities Transversal to Diagnostic Assessment and Post-Assessment *(continued)*
Guidance From the Key Player
44. Ensure that parents receive support from a key player until they are attended by workers and specialists in the public health educational services network or by specialists in private clinics.

Activities Transversal to the Entire Trajectory
Collaboration With Parents
45. Inform parents at all times of their child's situation, the clinical process, services offered to their child, etc.
46. Involve parents in the decision to refer their child to the appropriate support and intervention resources at all times, as early as the first concerns but also during and following assessment.
47. Ask parents for their consent at all times and for every new service offered or each new step taken.
Parental Support From a Key Person
48. Assign parents a navigator or a key player in the network to support them throughout the trajectory and consolidate their collaboration with workers and specialists involved in the trajectory.
Training and Support for Specialists Who Intervene With Children at All Times, From the Moment They Are Hired and on an Ongoing Basis
49. Participate in training and improvement activities (e.g., on the particularities of typical and atypical development, validated assessment tools, essential interpersonal skills for practice and intervention with families).
50. Access to indirect and direct supervision modalities (e.g., support from peer experts in diagnostic assessment).
51. Access to reference documents to support self-instruction (e.g., written documents).
Coordination and Consultation Between Various Service Providers (Including Early Childhood Settings, i.e., Environments That Include the Child or Offer Support to the Child and Family)
52. Establish collaboration mechanisms between clinical coordinators and managers.
53. Establish concertation mechanisms between programs offering services to young children, notably youth, mental health and intellectual disability, autism spectrum disorder, and physical disability programs.

Activities Transversal to the Entire Trajectory *(continued)*
Coordination and Consultation Between Various Service Providers (Including Early Childhood Settings, i.e., Environments That Include the Child or Offer Support to the Child and Family) *(continued)*
54. Implement access to information procedures that avoid duplication between previous and current assessments.
55. Implement an efficient information management system (e.g., for evaluating services, for monitoring cases).
56. Identify the various service providers (including early childhood settings) involved with the child.
57. Strengthen partnerships with the various service providers (e.g., communicate, establish agreements).
58. Convey required information to service providers, especially by producing written documents for that meet their needs (e.g., on eligibility criteria for assessment and services, on intervention strategies).
Continuous Evaluation of the Reference Trajectory in Diagnostic Assessment
59. Identify indicators allowing to evaluate the implementation of each dimension of the trajectory.
60. Collect data 1) that take into account quality indicators; 2) that are adapted to the objectives, phases, and contexts of implementation; and 3) continuously.
61. Present the results stemming from data collected from stakeholders (e.g., professionals, key players) to help revise their processes and adapt their practices.
62. Mobilize knowledge at all times to advertise the trajectory and practice changes related to process revisions (e.g., through infographics, newsletters, webinars, and communities of practice).

Appendix C

Activities Component of the Logic Model Presented to Advisory Committee Members in the Validation Phase

Transversal Activities
Involvement of Parents as Partners in the Diagnostic Services Trajectory
1. Ensure that parents can play an active role in the activities and steps of the trajectory, as early as the screening phase.
2. Inform parents at all times about their child's situation, the clinical process, services offered to their child (etc.) in a way that is accessible and understandable.
3. Ask parents for their consent at all times and for every new service offered or each new step taken.
4. Recognize parents' expertise and priorities by valuing their choices in the services offered for their child and family throughout the trajectory.
Accompaniment of the Child and Their Family From the Beginning of the Trajectory by a Key Player Who Ensures That They Are Directed to the Resources They Need
5. Assign the child and family a key player (e.g., patient navigator) who will accompany them throughout the trajectory, e.g. from screening through diagnostic assessment and onto specialized intervention services, in particular to 1) ensure that they have access to information, administrative supports (e.g. form completion), formal supports (e.g., needs assessment and intervention services); 2) ensure the proper coordination of the various services, physicians, and specialists involved in the child's and family's trajectory; and 3) ensure that the key player adopts the best practices to develop close relationships with the child and their family.
Organization of a Service Trajectory That is Accessible to All Children and Families Living in Quebec
6. Set up a system to ensure that at every stage of the trajectory child and family have access to support or intervention services at the right time and according to their needs.
7. Put in place procedures and mechanisms to reach all Quebec families and offer flexible services that adjust to family diversity.
Organization of a Service Trajectory That Meets the Evolving Needs of Children and Their Families
8. Implement a system that takes into account the child's response to interventions and supports in an iterative process throughout the child's trajectory and development.
Organization of a Service Trajectory That Allows Stakeholders to Carry Out Coordinated and Concerted Actions with Children and Their Families
9. Establish collaboration mechanisms between clinical coordinators and administrators.

Transversal Activities *(continued)*
Organization of a Service Trajectory That Allows Stakeholders to Carry Out Coordinated and Concerted Actions with Children and Their Families *(continued)*
10. Establish cooperation mechanisms between programs offering services to children, including youth programs and programs for mental health and intellectual disability, autism spectrum disorder, and physical disability.
11. Establish informational access procedures to avoid duplication between previous and current assessments.
12. Implement an efficient system for managing the information contained in the case file (e.g. to standardize the method of entering data collected by different service providers).
13. Identify, map, and make accessible information on regional service providers.
14. Strengthen the partnership between various providers (e.g., communicate, establish agreements).
15. Communicate necessary information to providers, including the development of written documentation (e.g., eligibility criteria for assessment and services, strategies for assessment and services, intervention strategies).
16. Put in place a system to ensure that each service provider has the necessary training to carry out their assigned role within the trajectory.
Evaluation of the Quality of the Implementation of the Trajectory in the Various Institutions and the Effects of its Implementation
17. Identify indicators to evaluate the implementation of each dimension of the trajectory.
18. Identify stakeholders for ongoing evaluation and include a representative of all stakeholders, including families, administrators, physicians and professionals.
19. Collect data that: 1) takes indicators into account; 2) is adapted to the objectives, phases, and implementation contexts; and is 3) ongoing.
20. Use the data collected and analyzed to revise processes and adapt stakeholder practices (e.g., physicians, specialists, key players).
21. Mobilize knowledge at all times in order to communicate the trajectory and practice changes associated with process revisions (e.g., through infographics, newsletters, webinars, communities of practice).

Pre-Assessment
Monitoring
22. Set up a universal monitoring system that: 1) reaches all children and families; 2) involves all caregivers working with children; 3) empowers caregivers to spot signs of developmental delays or atypical development; and 4) empowers caregivers to refer children to appropriate screening or diagnostic assessment services.
23. Supply providers with information on: 1) identifying children showing signs of developmental delays or atypicalities; 2) best practices for communicating concerns to parents; 3) mechanisms for requesting referrals for screening; and 4) mechanisms for keeping parents informed of ongoing and upcoming processes.
Referral for Screening or Diagnostic Assessment
24. Implement a referral system that allows providers to refer to diagnostic assessment services at any time.
25. Implement a one-stop access point for screening services to ensure a smoother processing of requests.
26. Ensure that all providers identify their criteria for referral to screening or diagnostic assessment services.
Screening
27. Set up a screening system: 1) for children for whom concerns have been raised by a parent or caregiver that; and 2) identifies children's needs and enables them to be rapidly referred to appropriate services and a more in-depth diagnostic assessment.
28. Develop a mechanism to communicate screening results and next steps to families.

Pre-Assessment and Diagnostic Assessment
Accessing Support and Services even Before a Formal Diagnosis is Made
29. Implement supports and services that meet the needs of children and their families that are accessible at all times: e.g., transdiagnostic support, parent coaching, psychological health coaching, information on financial support, community services, etc.
Listening to Parents and Their Concerns
30. Take into account parents' concerns about their child's development.

Diagnostic Assessment
Intake and Analysis of the Case File

31. Identify eligibility criteria for diagnostic assessment services, accounting for the fact that clinical profiles vary from child to child and may evolve during childhood.

32. Receive, read, and make an initial analysis of the case file, then acknowledge receipt to the referring environment.

33. Integrate information previously collected by the various providers prior to assessment.

34. Communicate information to parents, e.g., through a dashboard and flyers, on the assessment process and next steps (e.g., professionals involved, meeting procedures, assessment tools).

35. Conduct a telephone interview to confirm the reason for referral and the information (e.g., the source and nature of concerns).

36. Establish, during an interprofessional team meeting, the typical diagnostic assessment pathway for the child and their parents, based on the information gathered.

37. Discuss with parents how they can best play an active role during diagnostic assessment.

38. Plan the essential and optional stages of the entire diagnostic assessment process according the needs of the child and family.

39. When in the presence of one or more diagnoses that are not part of a neurodevelopmental disorder, neurological problems, genetic syndromes or physical health problems (etc.), set up a referral system to ensure more in-depth evaluation in the appropriate assessment services.

40. Involve physicians and other professionals in the diagnostic assessment, in accordance with the planning proposed in the typical diagnostic assessment pathway.

41. Ensure that diagnostic assessment relies on the clinical judgment of physicians and specialists to analyze and triangulate the various measures and sources of information.

42. Carry out a global assessment of all spheres of the child's development and document delays, developmental atypicalities, strengths, and interests.

43. Base diagnostic assessment on: 1) standardized, norm-referenced and non-standardized measures; 2) multiple data sources documenting the child’s functioning in their various living contexts (e.g., home, daycare, school) and according to various respondents (e.g., parents, educators, teachers); and 3) the child's response to various supports and interventions.

Diagnostic Assessment *(continued)*
Intake and Analysis of the Case File *(continued)*
44. Take into account: 1) family situations; 2) situations in other living environments; 3) the child's history (i.e., developmental history); and 4) the child's current clinical picture.
45. Link the assessment to: 1) the definitions and diagnostic criteria set out in the reference trajectory and 2) best practices in diagnostic assessment.
Functioning of the Interprofessional Team
46. Define and distinguish the mandates of physicians and specialists involved in professional corporations (orders/colleges), more specifically, fields of practice and reserved activities, in order to promote respect for their roles and responsibilities.
47. Structure a form of interprofessional collaboration that enables members of medical and professional groups to take collective action towards a common goal, through a process of communication, reflection, decision-making, intervention, and learning.
48. Organize regular meetings between physicians and specialists to discuss clinical impressions and draw diagnostic conclusions collectively.
49. Involve all physicians and specialists whose activities may contribute to the diagnostic assessment.
50. Adapt the level of interprofessional collaboration to the complexity of the situation.
Expertise and Continuing Education of Physicians and Specialists
51. Participate in training and development activities (e.g., on the particularities of typical and atypical development, validated assessment tools, the relational qualities essential to practice, support and intervention with children and families, partnerships with culturally and socially diverse children and families).
52. Have access to indirect and direct supervision (e.g., support from peers involved in diagnostic assessment, communities of practice, co-development practices).
53. Have access to reference documents to support self-training (e.g. written documents or video clips on the most common trajectories, the roles and mandates of physicians and specialists).
Diagnostic Findings
54. Consult parents before the assessment meeting to determine the support and modalities they will need when the diagnosis is disclosed.
55. Include in the assessment meeting: 1) disclosure of the diagnosis or clinical impressions; 2) presentation of the results; and 3) presentation of recommendations and information according to the needs of the child and their family and living environment.

Diagnostic Assessment *(continued)*

Diagnostic Findings *(continued)*

56. Ensure that the assessment meeting is conducted by physicians and specialists already involved in the assessment who apply best practices to develop and consolidate close relationships with parents.

57. Include the following in an assessment report: 1) assessments carried out by all physicians and specialists; and 2) Recommendations based on the needs of the child and their family and living environment.

58. Write the assessment report for parents: 1) to give them access to the diagnostic conclusions; and 2) to ensure that its contents are clear and adapted to their literacy level.

59. Support parents in sharing diagnostic findings or the assessment report, if they consent.

Diagnostic Assessment and Post-Assessment

Linking Diagnosis and Services

60. Ensure a link between the diagnosis made during the assessment and the services offered by the facility so that the diagnosis leads to services.

61. Ensure that the choice of individualized services is based on the results of the assessment.

62. Ensure that the necessary information from diagnostic assessment is communicated to specialized service providers so they can determine the intervention services best suited to the specific needs of the child and family.

Post-Assessment

Monitoring of Providers Who Provide Intervention Services to the Child

63. Refer to appropriate services following diagnostic assessment, according to the needs identified for the child and family and according to best clinical practices.

Monitoring of Providers Who Work With the Child

64. Follow up with caregivers who work with the child to inform them of the child's characteristics and provide them with intervention strategies.

Monitoring and Support for the Child, Parents, and Family

65. Following the assessment meeting, offer post-diagnosis follow-up meetings with the parents or child, depending on the nature of their needs, with flexible modalities (e.g., professionals involved, frequency and duration, individual or group, direct or indirect, virtual or face-to-face).

Post-Assessment *(continued)*	
Monitoring and Support for the Child, Parents, and Family *(continued)*	
66.	Ensure that post-diagnostic follow-up meetings: 1) allow parents or children or family to ask questions; 2) provide information adapted to the needs of children and families (e.g., on resources available in the community, the child's diagnosis and its characteristics, intervention strategies that parents and caregivers can apply, or specialized services); 3) support parents in completing forms for financial and material assistance (e.g., government allowances, grants from non-profit or charitable organizations); 4) accompany or refer the child and family to resources to positively support their mental health.
67.	Consider the nature of the needs and the context of the establishments (e.g., geographic, health, human resources) when choosing follow-up and support methods.

Appendix D

Representation of Iterative Reflective Work in Data Analysis

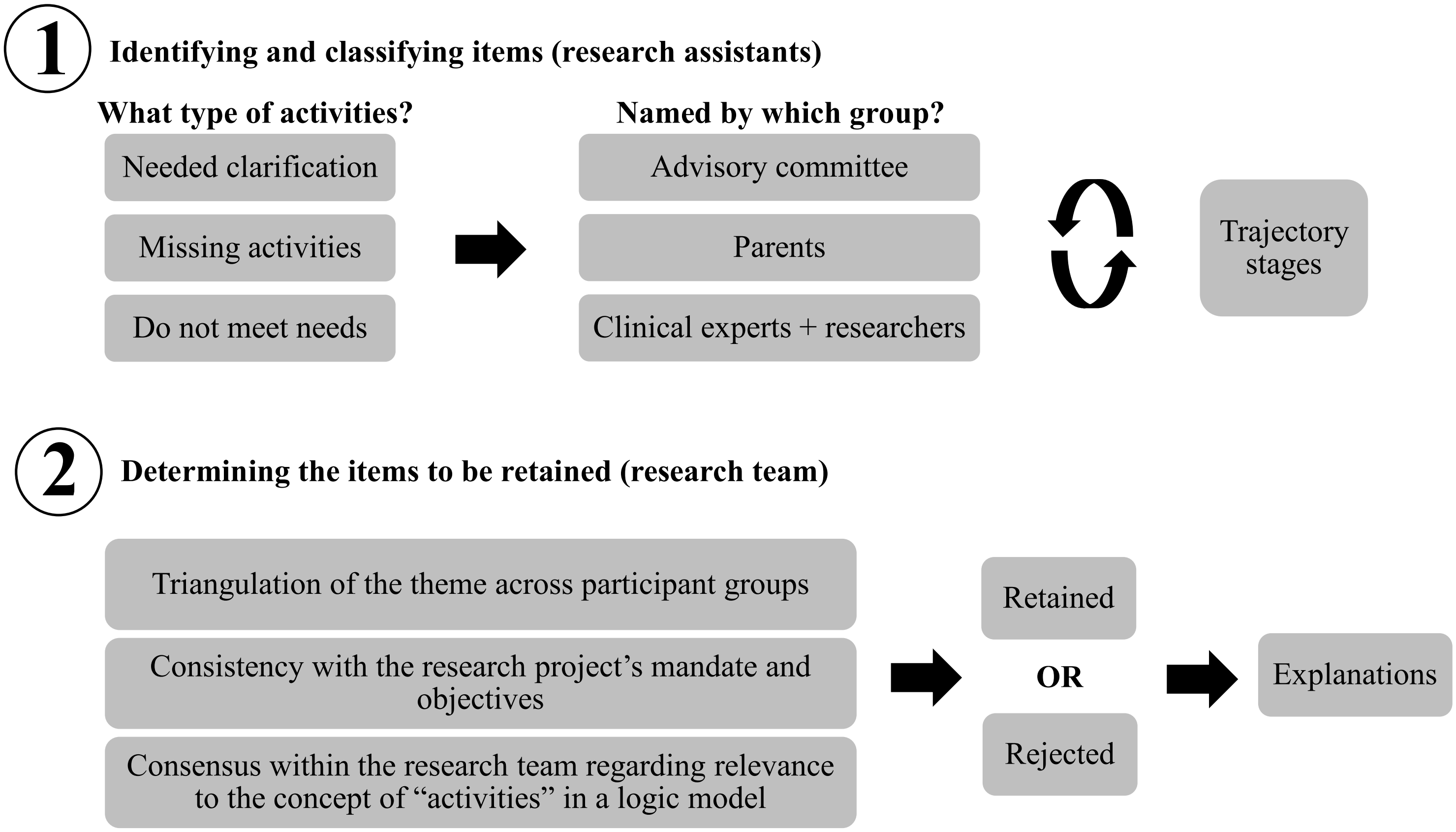

Appendix E

Themes Identified During the First Stage of Data Collection for Each Component of the Logic Model

Components	Themes
Problems	Insufficient capacity of institutional assessment and intervention services to meet increased demand (i.e., due to increased prevalence of developmental delays and atypicalities, neurodevelopmental conditions)<br><br>Difficulties in accessing early services for children, primarily due to longer waiting lists<br><br>Significant short- and long-term consequences for the child and family: development, school success, and quality of life<br><br>Family dissatisfaction with the accessibility, quality, continuity, adaptability of services and their adequacy to their needs
Objectives	Have a trajectory to organize diagnostic assessment services that takes into account best practices upstream and downstream of the assessment, as well as cross-cutting best practices<br><br>Create and consolidate partnerships between the various service providers involved in the trajectory<br><br>Take into account the experiences and needs of families in order to improve the services offered to them<br><br>Have a reference model that will support the deployment and updating of organizational trajectories within the institutions of the public health and social services network, thanks to continuous evaluation mechanisms
Target Groups	Primary target group, i.e., persons targeted by the services included in the trajectory: children under 7 years of age suspected of having a neurodevelopmental condition and their families<br><br>Secondary target group, i.e., healthcare providers and other practitioners involved in the diagnostic assessment service trajectory and their supervisors and administrators

Components	Themes
Groups Responsible	Administrators responsible for implementing the trajectory<br><br>Committees supporting the implementation and continuous improvement of the trajectory<br><br>Program evaluation expert teams responsible for the assessing the implementation of the trajectory and the effect of its implementation according to scientific criteria
Inputs	Financial resources<br><br>Human resources<br><br>Physical or virtual resources<br><br>Material or technological resources
Outputs	Source of the referral<br><br>Application status in the intake system<br><br>Capacity of the network<br><br>Clinical profiles of the children assessed<br><br>Completion of the processes within acceptable timeframes<br><br>Socio-demographic profiles of the children assessed and their parents<br><br>Views of stakeholders<br><br>Organization of services<br><br>Training opportunities for specialists involved in diagnostic assessment
Outcomes	Child- and family-centered approach (e.g., collaboration with parents, listening to needs, psychological support)<br><br>Referral process for screening or assessment (e.g., compliance with the process, clarity of information shared)<br><br>Diagnostic assessment process (e.g., compliance with the process, clarity of information shared, ways to facilitate parental involvement)

Components	Themes
Outcomes *(continued)*	Expertise of physicians and specialists in screening and diagnostic evaluation (e.g., consulting reference documents, using standardized assessment instruments with children)
	Training for physicians and specialists (e.g., access to high-quality, continuing education, support from peers)
	Quality of interprofessional collaboration (e.g., number of interprofessional team meetings deemed sufficient, involvement of multiple physicians and specialists in assessments as appropriate, coordinated planning of assessments)
	Diagnostic findings (e.g., understanding, appreciation, and validity of the report from parents' perspective)
	Post-diagnostic follow-up and support (e.g., opportunities for meetings and follow-up after diagnostic findings are announced, access to direct interventions for children and parent-mediated interventions)
	Coordination between different providers (e.g., support and intervention services, whether general or more specific, are planned, coordinated, and offered transversally)
	Ongoing evaluation and improvement process (e.g., establishment of procedures to evaluate the implementation of the trajectory and the effects of its implementation)
Influencing factors: Barriers	Transversal subcomponents
	Negative impact on access to services
	Negative impact on physicians and specialists’ expertise development
	Negative impact on interprofessional collaboration
	Negative impact on the availability, stability, and distribution of resources
	Negative impact on the continuity of services provided throughout the trajectory
	Negative impact on parents’ collaboration with physicians and specialists
	Negative impact on information-sharing between referrers, assessment services, and intervention services

Components	Themes
Influencing factors: Barriers *(continued)*	Negative impact on the evaluation of the implementation and effects of the trajectory<br><br>Negative impact on the optimal implementation of a reference trajectory on a provincial scale<br><br>Pre-assessment<br><br>Negative impact on the quality of screening<br><br>Diagnostic assessment<br><br>Negative impact on the quality of the assessment process<br><br>Negative impact on access to assessment services<br><br>Post-Assessment<br><br>Negative impact on the quality of post-assessment interventions<br><br>Negative impact on the effectiveness of post-assessment interventions
Influencing factors: Facilitators	Transversal subcomponents<br><br>Positive impact on access to services<br><br>Positive impact on physicians and specialists' expertise development<br><br>Positive impact on interprofessional collaboration<br><br>Positive impact on the availability, stability, and distribution of resources<br><br>Positive impact on the continuity of services provided throughout the trajectory<br><br>Positive impact on parents' collaboration with physicians and specialists<br><br>Positive impact on information-sharing between referrers, assessment services, and intervention services<br><br>Positive impact on the evaluation of the implementation and effects of the trajectory

Components	Themes
Influencing factors: Facilitators *(continued)*	Positive impact on the optimal implementation of a reference trajectory at a provincial scale<br><br>Pre-assessment<br><br>Positive impact on the quality of screening<br><br>Diagnostic Assessment<br><br>Positive impact on the quality of the assessment process<br><br>Post-assessment<br><br>Positive impact on the quality of assessment services<br><br>Positive impact on efficiency of assessment services

Appendix F

Subcomponents of Logic Model Activities of Trajectory

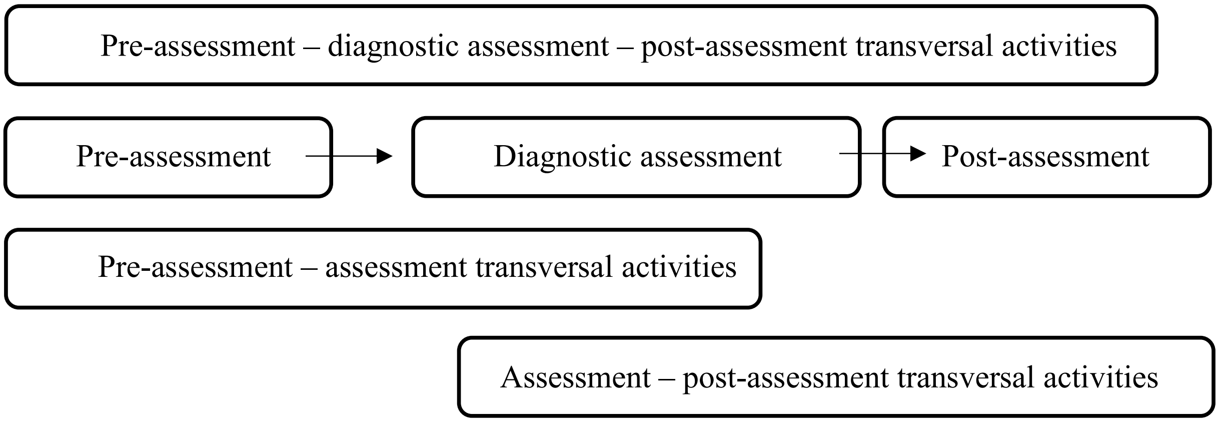

Appendix G

Barriers to the Completion of Logic Model Activities

Transversal Subcomponents
Negative Impact on Access to Services
1. Eligibility criteria that are overly restrictive (e.g., children under 6 years of age).
2. Excessive variability in eligibility criteria.
3. Requests that are not consistently centralized within a single reception system.
4. Lack of staff assigned to the reception system.
5. The challenges of prioritizing the high volume of requests.
6. Significant delays in obtaining services (and between services).
7. Difficulty comprehending the functioning of services, especially for families with complex migratory backgrounds.
8. The unequal distribution of resources, expecially in very large social-health regions where long-distance travel is frequently necessary.
9. Regional disparities in service provision, particularly in relation with the lack of experienced professionals in certain regions of the province of Quebec.
10. Lack of specialized services at the first level of diagnostic evaluation for children with suspected of having an autism spectrum disorder-ASD, intellectual disability-ID or global developmental delay-GDD.
11. Challenges in geographical accessibility (e.g., congestion problems congestion, limited parking spaces, lack of public transit) or difficulties in arranging transportation.
12. The lack of schedule flexibility to provide services.
Negative Impact on Physicians and Specialists' Expertise Development
13. Lack of training at the time of hiring and ongoing for physicians and professionals (e.g., on the diagnostic evaluation process).
14. Lack of mentoring or peer support.
15. The high degree of specialization among physicians and professionals in ASD, to the detriment of specialization in other diagnoses.
16. Lack of time to attend training sessions.

Transversal Subcomponents *(continued)*
Negative Impact on Physicians and Specialists' Expertise Development *(continued)*
17. Lack of knowledge, training and expertise of physicians and professionals in the process of diagnostic evaluation (compliance with DSM diagnostic criteria, consideration of risk and protective factors in diagnosis, differential diagnosis, diagnostic tools, etc.).
18. Frequent staff turnover, resulting in a loss of expertise within the teams.
Negative Impact on Interprofessional Collaboration
19. Lack of communication and coordination between physicians and professionals in the various departments and all providers.
20. A multi-professional work environment that doesn't facilitate interprofessional collaboration.
21. The challenges of adhering to Bill 21, which amends the Professional Code and other legislative provisions in the field of mental health and human relations.
22. The lack of official standards and practices, particularly when professionals are not regulated by an order.
Negative Impact on the Availability, Stability, and Distribution of Resources
23. Frequent staff turnover, leading to instability within teams (e.g., frequent changes in management without essential follow-up on certain files).
24. Rising demand without a corresponding increase in available resources.
25. Lack of time to plan and organize meetings, consultations and informal inter-professional discussions.
26. Lack of innovation and incentives to attract and retain qualified professionals in the network.
Negative Impact on the Continuity of Services Provided Throughout the Trajectory
27. Lack of coordination between departments.
28. Breakdowns in continuity of services, especially between referral and diagnosis.
29. The mandates of the various departments are unclear.
30. The administrative burden that limits organizational flexibility (e.g., extensive paperwork).
31. The mismatch between available services and the needs of children and their families.

Transversal Subcomponents *(continued)*
Negative Impact on the Continuity of Services Provided Throughout the Trajectory *(continued)*
32. Several waiting times along the trajectory.
33. The difficulty for parents to navigate the healthcare and social services system, to understand how the system operates, and to find their way within it.
34. The absence of a key player in the diagnostic assessment.
Negative Impact on Parents’ Collaboration With Physicians and Specialists
35. Information (e.g., reports, recommendations) provided in French or English only that limits understanding when one or both parents are allophones.
36. The role of certain physicians and professionals sometimes misunderstood by families.
37. The lack of interpersonal skills (sensitivity, empathy) that can sometimes be observed among certain physicians and professionals affects the quality of relationships with parents.
38. Lack of listening and consideration of parents' concerns by physicians and professionals.
39. The quantity and quality of information provided by physicians and professionals (e.g., information that is unclear or inadequate).
40. The gap between parents' and physicians' and professionals' perceptions regarding the child's development and needs (e.g., families are sometimes defensive).
41. The way in which physicians and professionals adjust to the emotional experience of families who are going through sensitive or vulnerable period.
42. Parents' confidence in physicians and professionals.
43. Lack of services to accompany and support families during and after the assessment.
44. Lack of information for parents about the various service providers and the coordination between them.
Negative Impact on Information-Sharing Between Referrers, Assessment Services, and Intervention Services
45. Lack of formalized communication systems with network and external partners.
46. Lack of resources to collaborate effectively, especially to follow up with referral organizations.

Transversal Subcomponents *(continued)*
Negative Impact on the Evaluation of the Implementation and Effects of the Trajectory
47. The issues regarding access to certain clinical-administrative data as well as the interpretation of these data (challenges related to the quality and validity of the data).
48. The challenges related to the capacity to meet management needs and clinico-administrative decision-making needs (in the development and evaluation of a trajectory, it is necessary to consider performance aspects, as well as contextual aspects, to give meaning to the data).
49. The fact of having clinical-administrative data management tools leads to heterogeneous and incomparable indicators in the different departments of the trajectory.
50. The often limited time makes it challenging to carry out a reliable and continuous implementation assessment.
51. The limited opportunities for meetings with individuals or groups leading the implementation, which restricts the evaluation and continuous improvement of the trajectory according to the needs of the environments and the evaluation team.
52. The perceptions of managers on one hand, and those of physicians and professionals on the other hand, which differ on certain aspects (e.g., differences regarding the importance placed on overall developmental assessment or clinical judgment, and the impact of these differences in perspective on practice).
Negative Impact on the Optimal Implementation of a Reference Trajectory on a Provincial Scale
53. Lack of sensitivity to local contexts in the healthcare sector which have their own level of complexity: implementation strategies that focus on the macro level may not produce the expected effects.
54. The lack of resources (e.g., grants, experienced professionals) and the instability of these resources, which limits the ability to implement the trajectory and offer screening, assessment and intervention services, as envisaged in the model.
55. Frequent changes in the strategic organization resources within the network (e.g., among managers) that limit the ability to implement services at full efficiency.
56. Resistance to changes in practices of certain stakeholders and professionals.
57. The persistent difficulties due to the Bill 10 adopted in 2015 (environments still need to adapt to this profound organizational transition)
58. The pressure on early assessment and intervention services in a context of increasing screening capacities.

Transversal Subcomponents *(continued)*
Negative Impact on the Optimal Implementation of a Reference Trajectory on a Provincial Scale *(continued)*
59. The challenges of harmonizing services across the territory, especially when comparing urban and rural areas, and when the territory is vast.
**Pre-Assessment**
Negative Impact on the Quality of Screening
60. The lack of systematic identification and screening of developmental delays in children.
61. The lack of a clear screening process for the entire province of Quebec.
62. Lack of appropriate, validated tools for detection and screening.
63. The limited capacity of the community to refer families for screening.
64. The lack of knowledge and training among physicians and professionals in identifying and screening delays (knowledge about typical and atypical developmental trajectories, identifying ASD in girls, using screening tools, etc.).
**Diagnostic Assessment**
Negative Impact on the Quality of the Assessment Process
65. The absence of a clear diagnostic evaluation process for the entire province of Quebec.
66. The absence of standards governing the practices of the involved in assessing neurodevelopmental disorders.
67. The over-specialization of professionals regarding ASD to the detriment of other diagnoses.
68. The multiplicity of physicians and professionals encountered to obtain a diagnosis.
69. Lack of materials, such as evaluation kits, to conduct assessments.
70. Lack of formal documentation to communicate information on evaluation trajectories (e.g., to parents, childcare parents, day-care centers, etc.).
71. The fact that the child's potential for growth and maturity is difficult to consider in a diagnostic evaluation process that must be carried out over a limited period.
72. Challenges in terms of the validity of clinical criteria for assessing certain profiles (e.g., ASD in young girls).

Diagnostic Assessment *(continued)*
Negative Impact on the Quality of the Assessment Process *(continued)*
73. Lack of flexibility in the prescribed assessment framework for the evaluation of complex clinical cases requiring a longer and better adapted assessment (e.g., addition of observations in a day-care setting, complementary occupational therapy assessment).
74. Duplication of diagnostic assessment activities carried out by different professionals within the trajectory.
75. The difficulty of securing the assessment process when several professionals are involved, making interprofessional work ineffective.
76. The time it takes to obtain a diagnosis and the length evaluation process.
77. Parents' lack of availability (e.g., parents who don't have enough time or whose commitment is weaker), which can affect the completion of the steps planned in the diagnostic evaluation process, as well as the duration and quality of the process.
Negative Impact on Access to Assessment Services
78. Access to evaluation services in certain geographical contexts (physical distance from families causing absenteeism, delays, and wait times in evaluation).
79. Access to assessment services limited by the lack of systematization of monitoring and screening mechanisms.
80. Lack of adequate physical environments to offer services (e.g., assessment rooms too small and too few in number).
Post-Assessment
Negative Impact on the Quality of Post-Assessment Interventions
81. Lack of adapted services to meet children's needs following diagnostic findings.
82. The lack of systematization in the implementation of individualized service plans.
83. The absence or lack of post-diagnostic follow-up and services for children with GDD or ID compared to children with ASD (e.g., lack of information on available treatments, services and formal support, and on navigating the healthcare system).
84. Lack of guidelines for directing families to appropriate resources following diagnosis: what post-diagnostic services to be offered to children and parents.
85. Lack of recommendations and information for children with milder difficulties.

Post-Assessment *(continued)*
Negative Impact on the Efficiency of Post-Assessment Interventions
86. Waiting times between services.
87. The late age at which children are assessed makes it impossible to offer early intervention before they start school.

Appendix H

Facilitators of the Completion of Logic Model Activities

Transversal Subcomponents
Positive Impact on Access to Services
1. The physical accessibility of assessment and intervention for families (e.g., public transit).
2. The central urban location of clinical environments.
3. Premises accessible to people with reduced mobility.
4. A range of services that takes family availability into account.
5. A service offering that considers linguistic barriers.
6. Accessibility to assessment and intervention services for all children and families who need them, irrespective of criteria that might disadvantage certain profiles in NDC, and according to the needs of children and their families.
7. Harmonization of access and quality of services across the regions.
Positive Impact on Physicians and Specialists' Expertise Development
8. The ability of physicians and professionals to adapt and integrate best practices.
9. The ability to work interprofessionally.
10. Physicians' and professionals' perception that they are being support in their work (e.g., advice, training).
11. Access to support for physicians and professionals, as needed.
12. The presence of an advisory committee (clinical experts) to support the team.
13. Access to a training program upon hiring as well as to ongoing training.
14. The knowledge of processes by physicians and professionals through training courses or guides.
Positive Impact on Interprofessional Collaboration
15. Understanding of the roles of each team member and seamless alignment with these roles and responsibilities by the teams.
16. The implementation of means to facilitate communication between physicians and professionals.

Transversal Subcomponents *(continued)*
Positive Impact on Interprofessional Collaboration *(continued)*
17. Interprofessional collaboration and role recognition.
18. Considering Bill 21 on reserved activities within the diagnostic evaluation trajectory.
19. The implementation of measures to ensure the retention expertise within the organization.
20. The implementation of mechanisms to ensure a good team spirit and a good teamwork climate.
21. Established clinical and team operating processes in a clear and fluid manner.
Positive Impact on the Availability, Stability, and Distribution of Resources
22. Networking to increase resources and assessment capabilities with other clinics and private settings, other professionals (e.g., speech therapists, child psychiatrists), or with universities (to attract students).
Positive Impact on the Continuity of Services Provided Throughout the Trajectory
23. The presence of the key worker promotes continuity and service navigation.
24. The inclusion of several program-services under a single same management.
25. The use of clinical-administrative management tools (e.g., computer platform) to facilitate case tracking.
Positive Impact on Parents' Collaboration With Physicians and Specialists
26. The ability to build trust with families through the interpersonal skills of the interprofessional team.
27. Developing interpersonal skills: empathy, listening, sensitivity, tolerance, patience, humility, respect, empowerment of families.
28. Training for physicians and professionals on how to develop families' trust in a variety of contexts (e.g. multiculturalism).
29. The time needed to put these values into practice.
Positive Impact on Information-Sharing Between Referrers, Assessment Services, and Intervention Services
30. Close collaboration between managers and clinical supervisors (directors or heads of departments of service providers), and their involvement in all stages of the diagnostic process.

Transversal Subcomponents *(continued)*
Positive Impact on Information-Sharing Between Referrers, Assessment Services, and Intervention Services *(continued)*
31. Consultation between departments, clinical coordinators and managers or department heads.
32. A well-established communication process with referral organizations.
33. Cross-sector collaboration and partnerships with providers.
34. Reducing the administrative burden and using tools that facilitate follow-up (e.g., by using recognized software, whether for scheduling assessment meetings, tracking the progress of assessments or keeping records).
Positive Impact on the Evaluation of the Implementation and Effects of the Trajectory
35. The mixed quantitative/qualitative approach (and data triangulation).
36. The implementation of iterative feedback processes, including recommendations to improve trajectory quality throughout its implementation.
37. Recommendations based on best practices and contextual realities.
38. The use of various collection tools.
39. Gathering data from the viewpoints of various groups of participants.
40. The implementation of a system for continuous evaluation of implementation, coupled with a system for continuous improvement of service quality based on this evaluation.
41. Taking parents' views into account when improving services.
42. Implementing a bidirectional clinical-research sharing to compare best practices documented in literature with those observed in the field.
Positive Impact on the Optimal Implementation of a Reference Trajectory at a Provincial Scale
43. Identifying the right people to drive change.
44. Preparing players for change.
45. Optimizing the potential of players by offering them the support and resources.
46. Close collaboration with parents in services throughout the trajectory.

Pre-Assessment
Positive Impact on the Quality of Screening
47. Quality of surveillance services and upstream screening.

Diagnostic Assessment
Positive Impact on the Quality of the Assessment Process
48. Considering the reason for consultation and information from referring environments (e.g., screening) that will influence the evaluation trajectory.
49. Planned processes that remain flexible to adapt to the needs of children with more complex profiles and needs (e.g., the use of several pre-established assessment processes with formalized steps, by profession and diagnosis).
50. A diagnostic assessment based on multiple sources of information (e.g., standardized assessment tools, professional judgment, developmental history, risk and protective factors).
51. A diagnostic evaluation process that favors interprofessional collaboration (by more than one professional and physicians) but avoids the multiplicity of people families meet and the repetition of information requested.
52. Diagnostic evaluation, with a focus on best practices.
53. Information available and transmitted to parents explaining the various stages of the diagnostic evaluation.
54. Identifying the child's strengths and needs.
55. The clinical skills, professionalism and interpersonal skills of those carrying out the assessment and diagnosis.
56. The presence of a complete clinical team who can work interprofessionally and who have mastered the diagnostic evaluation process and tools.
57. The types of information shared in the diagnostic announcement (e.g., the child's strengths and interests or possible services).
58. Parents are given the space they need to ask questions and obtain clear, accurate information throughout the assessment process and when diagnostic conclusions are announced.
59. Increased consultation between doctors and professionals when the diagnosis is difficult to make.

Diagnostic Assessment *(continued)*
Positive Impact on the Quality of the Assessment Process *(continued)*
60. From team consultation to final decision.
61. The participation of physicians and other professionals in the assessment process, in accordance with the mandate entrusted to them and with respect for interprofessional work.
62. Obtaining a final diagnosis and assessment report to understand and continue the process of accessing services.

Post-Assessment
Positive Impact on the Quality of Post-Assessment Interventions
63. Referring families to the appropriate resources after diagnosis.
64. Collaboration between the various providers involved with children and families in the service trajectory.
65. Information on the types of services that can be offered to families.
66. Preparation of a report including recommendations for individualized intervention.
67. The implementation of structured and continuous interventions.
68. The implementation of direct child-centered interventions (individual or group).
69. The implementation of specialized interventions adapted to the needs of the child.
70. The provision of individual and group intervention modalities throughout the trajectory, particularly during the waiting period for assessment services.
Positive Impact on Efficiency of Post-Assessment Services
71. The implementation of intervention as soon as the diagnosis is made to avoid compromising the child's development and family balance.
72. Setting up an individualized service plan.
73. Service provision that includes varied, direct and indirect, structured interventions, whether they are individual or group-based.

Appendix I

General Comments by Stakeholders

Themes	Stakeholder Comments
Comprehensive	Expert: Sounds like a complete model<br><br>A member of the advisory committee: So we really need to find a way to communicate this better, to simplify things, because there are some doctors who will decide to refer elsewhere because it just seems, like, too complicated. Where do I start? What do I do? It's important to communicate what Agir tôt [Act Early] is, what the diagnostic pathway is, the difference between screening and surveillance. They've started to do that, you know? Agir tôt [Act Early], they have a website for healthcare personnel, but people still need to know that it exists. So, you know, let's get it out there, let's communicate the trajectory model more widely.
Consistent with the Act Early program	Expert: I thought they were interesting, and then, basically, they're similar to what's being proposed with the Agir tôt [Act Early] program. So, basically, what's emerging with us. So I was reading what was happening in terms of monitoring, I wrote for myself that it was adjusted to the reality, and to the wishes expressed by the Ministry as well.
Solution-Oriented	A member of the advisory committee: When you're in health promotion, you're in the positive. We don't look for problems, we don't try to avoid them, whereas a medical approach is to look for problems in order to find solutions. And that's what you're doing: you're looking for problems, and when you've found them, you're going to provide the answers.

Themes	Stakeholder Comments
Integrates Multiple Expertises and Viewpoints	Expert: For me, it also demonstrates a profound knowledge based on the literature, but also experiential intervention. It reflects expertise and years of experience. Probably years of having listened to families' concerns and so on.<br><br>A member of the advisory committee: Then everything else made sense to me. It was really, really good. Consolidating partnerships. Why did I put a star here? It had to be-, I put a star to identify the different service providers, consolidate partnerships with the different providers, transmit the necessary information to the service providers. Well, I guess I liked that one. I didn't write any more notes.
Child-Centered within an Ecosystemic Perspective	Expert: It's clear how you present it in your model. It's not compartmentalized, it's really interrelated, and we can see that the child is really at the heart of the concern. For me, it's well presented.<br><br>A member of the advisory committee: I really liked the fact that there are key places that need to be made particularly aware, informed of-, for example, daycares or schools too, you know.<br><br>Expert: I think I was impressed, rather, that there were support or communication benchmarks that were established in this first part, so, if we detect something, we make sure that the various stakeholders are aware of it. So, that was… Basically, I found it very complete.<br><br>Expert: It seemed that there were moments of communication between the systems, so to break down, perhaps, the silos of the educational system, family system, the diagnostic screening system and… So, it was good to see that there was a concern to break down the silos.

Themes	Stakeholder Comments
Supports Parents and Promotes their Involvement	A member of the advisory committee: It's an important aspect, but I think that in the document you've given us, it's very well described, all the importance we have to give, and the follow-up and accompaniment we have to do, especially for the parents.<br><br>Expert: What I find interesting in your model is that the parent – as much during the assessment as during prep[-assessment], It's not: we take charge of your child, we see what's going on, we give you updates, you know? You have to integrate, you have to participate.<br><br>Expert: I really enjoyed reading about post-diagnostic support and services, both for families and for specialists working at the clinic.

Appendix J

Barriers and Facilitators Associated With the Implementation of the Trajectory

Themes	Facilitators	Barriers
Administrative support	Presence of an administrative officer	The addition of another procedure can be administratively cumbersome and add to the pitfalls (e.g., requiring written consent).<br>Relieve clinicians of administrative tasks and consider administrative support.
Presence and role of key players	Assigning to the child and their family a coordinator who will support them according to their needs.	
Availability of human and physical resources (in particular, digital technology)	Having sufficient manpower to implement the anticipated trajectory.<br>The deployment of the digital health records is essential to the success of this type of coordination and collaboration.<br><br>We need to move quickly to a digital solution.	Presence of a labor shortage.<br><br>Difficulty in filling positions, high staff turnover, and sometimes widely disparate levels of training and experience.<br><br>Intra- and extra-organization collaboration is needed, but difficult because of administrative barriers, systems that don't communicate and outdated practices (e.g., fax).<br>Institutional archives seem reluctant to move into the digital age, and we're overrun by forms and paper.<br>For children who don't have a family doctor or pediatrician, it becomes very difficult to refer to a medical service or doctor when you're a healthcare professional.
Assessment and intervention approach based on needs rather than diagnosis	Maintaining a certain flexibility to adjust the intervention to needs.	Waiting lists and performance challenges mean we have to get straight to the point.<br><br>Most programs are based on diagnosis or needs, so it's not easy to access services if you don't exactly fit the criteria.

Themes	Facilitators	Barriers
Coordination of public and private services	Good communication between the private and public sectors.	
Promoting functioning and providing information on the diagnostic trajectory (particularly among parents, but also health, social service, and education professionals)	Computerized solutions offer parents the possibility of responding, reading, or consulting at a time that suits them, with the help of a trusted person to translate if necessary. Of course, the possibility of contacting a specialist should always be included in the options available.	Difficulty reaching parents in real time, because they work, have more than one child, can't respond in a quiet environment, don't speak English or French, or need the support of another family member to respond.
Promoting reserved acts regulated i.e., acts for which practice is by professional orders/colleges		Assessment tools are incorrectly assumed to be reserved for a specialist, or that the expertise of one medical specialty is required rather than another to make a diagnosis.<br><br>Lack of understanding of which tools are reserved and which are not, and this is where work needs to be done. It's true that some specialists have wrongly claimed that certain tools were reserved for them. For example, some psychologists said that psychoeducators couldn't work with ADOS [Autism Diagnostic Observation Schedule], which was untrue. However, I'm not sure that this is our mandate, but rather that specialists should refer to their professional corporations and read the standards for tool use.
Continuing education and development of professional expertise		In reality, there is very little advanced training organized by the network.

Appendix K

Agreement of the Advisory Committee Members on the Components of the Logic Model for a Diagnostic Services Trajectory

Percentage of Agreement of Each Member for Activities	P1	P2	P3	P4	P5	P6	P7	P8	P9	P10	***M* by activity**
Transversal Component	100.0	87.7	100.0	100.0	100.0	100.0	95.2	100.0	100.0	100.0	**98.1**
Pre-Assessment Component	85.7	100.0	100.0	100.0	100.0	100.0	85.7	100.0	100.0	100.0	**97.1**
Pre-Assessment Transversal Activities Component	100.0	100.0	100.0	100.0	100.0	100.0	100.0	100.0	100.0	100.0	**100.0**
Diagnostic Assessment Component	100.0	89.2	100.0	100.0	100.0	100.0	75.9	96.6	100.0	93.1	**95.2**
Transversal Post-Assessment Activities Component	100.0	100.0	66.7	100.0	100.0	100.0	100.0	100.0	66.7	66.7	**90.0**
Post-Assessment Component	100.0	100.0	100.0	100.0	100.0	100.0	100.0	100.0	100.0	100.0	**100.0**
M by member	98.5	86.6	98.5	100.0	100.0	100.0	86.6	98.5	98.5	95.5	**96.6**

Appendix L

Activities of the Logic Model

<table>
<tr><th rowspan="5">ACTIVITIES</th><th>PRE-ASSESSMENT</th><th>DIAGNOSTIC ASSESSMENT</th><th>POST-ASSESSMENT</th></tr>
<tr><td colspan="3">1. Involvement of parents as partners in the service trajectory;
2. Support for the child and their family, from the start, by a key player who ensures that they are directed to the individualized resources they need;
3. Access for all children and families living in Quebec;
4. Adaptation to the evolving needs of children and their families;
5. Support for stakeholders to carry out coordinated and concerted actions with children and their families;
6. Evaluation of the quality and effects of the implementation of the trajectory across the various institutions</td></tr>
<tr><td colspan="2">1. Accessing support and services prior to a formal diagnosis is made;
2. Listening to parents and their concerns.</td><td></td></tr>
<tr><td></td><td colspan="2">1. Aligning the diagnosis with support and intervention services.</td></tr>
<tr><td>1. Developmental monitoring;
2. Referral for screening or diagnostic assessment;
3. Screening of children for whom concerns have been raised.</td><td>1. Intake and analysis of the case to establish the assessment pathway;
2. Evaluation process that includes various methods and is based on best practices
3. Working in an interprofessional team;
4. Expertise and continuing education of physicians and specialists of the interprofessional team;
5. Diagnostic conclusion and support for parents.</td><td>1. Follow up with providers who offer specialized services to the child;
2. Follow up with providers who work with the child;
3. Follow up and support for the child and their family.</td></tr>
</table>
